# How do previously inactive individuals restructure their time to ‘fit in’ morning or evening exercise: a randomized controlled trial

**DOI:** 10.1007/s10865-022-00370-x

**Published:** 2022-11-03

**Authors:** Paige G. Brooker, Sjaan R. Gomersall, Neil A. King, Nicholas F. McMahon, Michael D. Leveritt

**Affiliations:** 1 School of Human Movement and Nutrition Sciences, Faculty of Health and Behavioural Sciences, The University of Queensland, St. Lucia, QLD Australia; 2 School of Health and Rehabilitation Sciences, Faculty of Health and Behavioural Sciences, The University of Queensland, St. Lucia, QLD Australia; 3 School of Exercise and Nutrition Sciences, Faculty of Health, Queensland University of Technology, Kelvin Grove, QLD Australia

**Keywords:** Exercise time-of-day, Use of time, Randomized controlled trial, Physical activity, Obesity

## Abstract

The objective of this study was to investigate changes in sedentary and active behaviors when previously inactive adults start exercising in the morning or evening. One-hundred adults with overweight or obesity (BMI ≥ 25 kg/m^2^) were recruited for a 12-week intervention and randomized to one of three groups: (i) morning exercise (AMEx; 0600–0900); (ii) evening exercise (PMEx; 1600–1900); or (iii) waitlist control. AMEx and PMEx were prescribed self-paced aerobic exercise to achieve a weekly total of 250 min via a combination of supervised and unsupervised training. Sedentary and active behavior times were measured at baseline, mid- and post-intervention using the multimedia activity recall for children and adults. Time spent engaging in physical activity was significantly increased from baseline at both mid- (+ 14–22 min·day^−1^) and post-intervention (+ 12–19 min·day^−1^), for AMEx and PMEx. At 12-weeks, participants in both morning and evening exercise groups reported increased time spent Sleeping (+ 36 and + 20 min·day^−1^, respecitively), and reduced time spent watching TV/playing videogames (− 32 and − 25 min·day^−1^, respectively). In response to an exercise stimulus, previously inactive adults make encouraging modifications in how they use their time, and the patterns of change are similar with morning and evening exercise.

**Supplementary Information:**

The online version contains supplementary material available at 10.1007/s10865-022-00370-x.

## Introduction

The health benefits of exercise for reducing the risk of non-communicable diseases such as cardiovascular disease, type II diabetes and some cancers, are well documented (Centers for Disease Control & Prevention, 2018). Despite this, 44.5% of Australian adults are either inactive (14.8%), or have low levels of physical activity (29.7%) (Australian Government Department of Health, 2017), often attributed to a self-reported lack of time (Sallis & Hovell, 1990).

The number of hours in a day is fixed and finite, distributed between obligatory time (such as time spent working, sleeping, domestic activities, commuting or eating) and discretionary time (the remaining ‘free’ time) (Goodin et al., 2002; Mekary et al., 2009). Some activities are seemingly ‘inelastic’ and require a fixed amount of time (such as work commitments) while others seem to be ‘elastic’ (such as sleep, screen time, eating, and exercise) (Olds et al., 2012). Physical activity researchers have started to consider individuals’ whole-of-day activity patterns to help understand how people use their time (Baere et al., 2015; Gomersall et al., 2014), and therefore, to identify timeframes when new activities, such as exercise, can realistically be incorporated and sustained. However, to ‘make time’ for a new exercise program, time must be drawn from another activity/activities. These activity ‘modifications’ can have important health consequences; i.e., the activities that are displaced may enhance, or mitigate the positive effects of exercise (Chastin et al., 2015; Gomersall et al., 2014; Mekary et al., 2009, 2013). Gomersall and colleagues investigated the changes in use-of-time across a 6-week physical activity intervention in a sample of 129 insufficiently active adults (Gomersall et al., 2014). The researchers found that time to accommodate increased physical activity predominantly displaced time spent watching television, suggesting an encouraging activity modification. Eleven participants dropped out of the study due to ‘being unable to make the time commitment’. When considering individual’s social, work and family commitments, previously inactive individuals may try to accommodate exercise at a time-of-day which is not sustainable long-term.

To improve compliance and adherence to exercise, the concept of temporal consistency has been proposed. Regularly performing an activity at a specific time, may be important for long-term adherence as it aids in creating a ‘protected time’ for exercise habits (Kaushal & Rhodes, 2015; Rhodes & Bruijn, 2010). Morning and evening (before and after work) are key windows of opportunity to incorporate exercise (Bailey & Jung, 2014; Brooker et al., 2021; Schumacher et al., 2019). The few studies which have investigated the influence of exercise time-of-day on physical activity participation / exercise adherence report mixed findings. Burn et al. (Burn et al., 2017) reported greater compliance in the ‘after-work’ (i.e., afternoon/evening) group compared with the ‘in-work’ (i.e., at lunchtime) group in their 40-day work-place physical activity intervention (70% v. 26%, respectively). Other studies have compared morning and evening periods; some favour morning (Bailey & Jung, 2014; Bond et al., 2017), and others report no difference in rates of exercise adherence between morning and evening periods (Blasio et al., 2010; Brooker et al., 2019; in press; Creasy et al., 2022). While there is agreement that regular exercise plays an important role in improving general health and maintaining energy balance, there remains a distinct lack of evidence regarding an optimal time-of-day for exercise to maximize compliance.

The activity swaps in response to morning or evening exercise are not understood, and are likely to be different. For example, a person who embarks on a new morning exercise program may forego their recreational walk (Sahlqvist et al., 2013).This type of displacement could result in a negligible net increase in overall physical activity and thus, energy expenditure, attenuating the benefits of the added exercise. Conversely, the addition of morning exercise to an individual’s existing routine may lead to a displacement in sleep, whereby individuals wake up earlier for exercise. This hypothesis is supported by the work by Gomersall et al. (2014) who found a trend for reduced sleep (− 30–− 41 min·day^−1^) when individuals were prescribed 150 and 300 min of moderate-vigorous physical activity per week for six weeks, compared with a control group of usual activity, suggesting that individuals may substitute sleep to fit in exercise. If sleep is displaced as a result of increased activity, the positive benefits of exercise may be attenuated, if baseline sleep duration is low (Chaput, 2014). Another possible scenario, for example as could be seen with the addition of a new evening exercise program, is time could be drawn from sedentary activities such as watching television (Olds et al., 2011), a displacement which would likely result in an overall increase in physical activity (Dunstan et al., 2010). There is observational and experimental evidence to support the hypothesis that changing patterns of time use are likely to have flow on effects for health (Chastin et al., 2015; Gomersall et al., 2014). For example, in a longitudinal sample of 4,558 adult females, Mekary and colleagues studied the isotemporal substitution effects of physical activity and sedentary behavior on weight status (Mekary et al., 2009). They found that changes in weight status were dependent on what activity was displaced by exercise in the overall time budget; an increase of 30 min·day^−1^ resulted in a weight loss of 1.6 kg if it displaced a brisk walk, compared to a weight loss of 3.7 kg if it displaced TV viewing.However, there are no studies which have compared the impact of exercise time-of-day on patterns of time use.

Therefore, the objective of this study was to investigate how previously inactive adults restructure their time when they undertake morning or evening exercise. This study was conducted within a larger randomized controlled trial aimed at investigating the influence of time-of-day of exercise on cardiometabolic health (Brooker et al., 2019; in press).

## Methods

This study was registered with the Australian New Zealand Clinical Trials Registry (*blind for peer review*) and approved by the Bellberry Human Research Ethics Committee (HREC2016-02-130). Informed consent was obtained from all individual participants included in this study. All procedures, including the informed consent process, were conducted in accordance with the ethical standards of the responsible committee on human experimentation (institutional and national) and with the Helsinki Declaration of 1975, as revised in 2000. The intervention is reported in accordance with CONSORT (See Supplementary Material).

Participants were recruited from the local community and metropolitan universities via electronic media and print advertising. Interested individuals were screened for eligibility by web-based or telephone survey, which included stage one of the Adult Pre-exercise Screening System, developed by Exercise and Sports Science Australia (Exercise & Sports Science Australia, 2012). To be included in the study, individuals were required to be: (i) insufficiently active (accumulating < 150 min of moderate-vigorous physical activity per week, by self-report); (ii) overweight or obese (body mass index (BMI) ≥ 25 kg/m^2^); and (iii) weight stable in the previous three months (± 3 kg by self-report). Individuals were considered ineligible if they: (i) were pregnant, or had plans to become pregnant over the course of the study; (ii) participated in shift work; (iii) were currently participating in a weight loss program; or (iv) were using any medication or supplements that would affect food intake, appetite or physical activity levels, weight loss, or metabolism. Individuals deemed eligible and who obtained medical clearance (as required based on their responses to the Adult Pre-exercise Screening System), or did not require it, attended the laboratory for their baseline assessment. Ineligible individuals, and those who failed to obtain medical clearance were excluded from the study.

This study used a three-armed, randomized controlled trial design, with a 12-week lifestyle intervention. Following baseline testing, participants were randomized into one of two intervention groups, or a waitlist control group (CON) at a 2:2:1 ratio using permuted block randomisation with multiple, randomized block sizes by a researcher external to the study. Due to the nature of the intervention, participant blinding was not possible. Participants allocated to CON were asked to continue with their day-to-day activities and were offered the exercise program after all formal testing was completed. The two intervention conditions comprised of a 12-week exercise program in which participants were prescribed a minimum of 250 min of moderate-vigorous exercise per week; the dose of exercise recommended by the American College of Sports Medicine to elicit clinically significant weight loss (Donnelly et al., 2009). Participants randomized to the morning group (AMEx) were required to exercise between 0600–0900, and 1600–1900 for those in the evening group (PMEx). The time periods were chosen to coincide with diurnal hormone patterns, and for convenience based on when most people could accommodate exercise (i.e., before or after work), to enhance any physiological adaptations which may occur as a result of exercising at either time-of-day, and maximize adherence to the sessions (Brooker et al., 2021).

The exercise program included both supervised and unsupervised exercise sessions. Participants completed an initial four-week supervised exercise training phase, of five 50 min sessions per week. Over the remaining eight weeks, exercise sessions were tapered by one session per fortnight until two sessions per week was reached, which was then maintained for the remainder of the intervention. Supervised sessions consisted of self-paced brisk-walking or running on a treadmill. All supervised exercise sessions were conducted at the School of Human Movement and Nutrition Sciences at The University of Queensland, St. Lucia, Australia. The secondary component of the intervention involved several constituents of theoretical approaches to encourage behavior change. Informational and behavioral approaches were the focus of the intervention. Strategies used to enhance behavior change are outlined in Supplementary Table A1.

Self-reported use-of-time was measured using the adult version of the MARCA; a computerised 24-h recall tool which asks participants to recall all activities from their previous day (midnight to midnight), in increments as small as five minutes. The MARCA was administered by a computer-assisted telephone interview in an open-ended format, using meal times as reference points in a segmented day format, on two occasions approximately one week apart. Each time, two consecutive days were recalled; therefore, at each measurement participants were asked to recall four days (two weekend and two weekdays). Where possible, recalled days were kept consistent between measurement occasions. During the recall, the interviewer selected an appropriate activity from a list of over 500 activities, based on an expanded version of the Ainsworth compendium (Ainsworth et al., 2011).

Time use profiles were cleaned and checked; firstly, any activities entered as ‘other’ were identified and replaced with a similar activity (based on body position and energy expenditure) from the compendium; next, any time use profiles with missing data (< 24-h, or 1440 min) were identified and excluded; finally, participants with < 2 time use profiles recorded or did not include one weekend day per assessment period were excluded. To determine time use, participants’ individual time use profiles were established by calculating the time spent in major ‘activity sets’ and collapsed hierarchically into domains based on similarity and to preserve comparability with previous studies(Gomersall et al., 2014). Eleven mutually exclusive ‘Superdomains’ were used; physical activity, computer, active transport, passive transport, quiet time, self-care, socio-cultural, work/study, chores, sleep, and TV/Videogames (previously established in greater detail (Gomersall et al., 2010), and described in Supplementary Table A2).

All data were analysed using the statistical package for social sciences (SPSS) version 25 (IBM, New York, USA). Statistical significance was set at an alpha of *p* < 0.05. All results are reported as Mean (Standard Deviation), unless specified otherwise. Linear mixed modelling with fixed and random effects was used to assess changes over time and differences among groups, estimated by a restricted maximum likelihood algorithm. Group (AMEx, PMEx, CON), time (0, 6, 12), and group × time interaction were treated as fixed factors; participants were treated as a random factor with individual intercepts. Model residuals were formally assessed for normality by use of the Shapiro–Wilk test and visual inspection of histogram plots. Fishers Least Significant Difference test was used for posthoc analyses to compare mean changes in time use between groups at each assessment period. A priori power calculations determined that with an alpha of 0.05 and 80% power, we could detect a small effect size (Cohen’s *d* = 0.3) with a sample of 95 (*n* = 38 for intervention groups; n = 19 for CON).

## Results

One-hundred participants were randomly allocated to AMEx (n = 40), PMEx (n = 40), or CON (n = 20) groups. Eighty-two participants completed the intervention. Reasons for drop-out were due to personal, work or family reasons (n = 12), being unable to make the time commitment (n = 4) or medical reasons (n = 2). Participants were recruited on a rolling basis from June 2016 to May 2017. Follow-up testing was completed in August 2017.

**Fig. 1:**
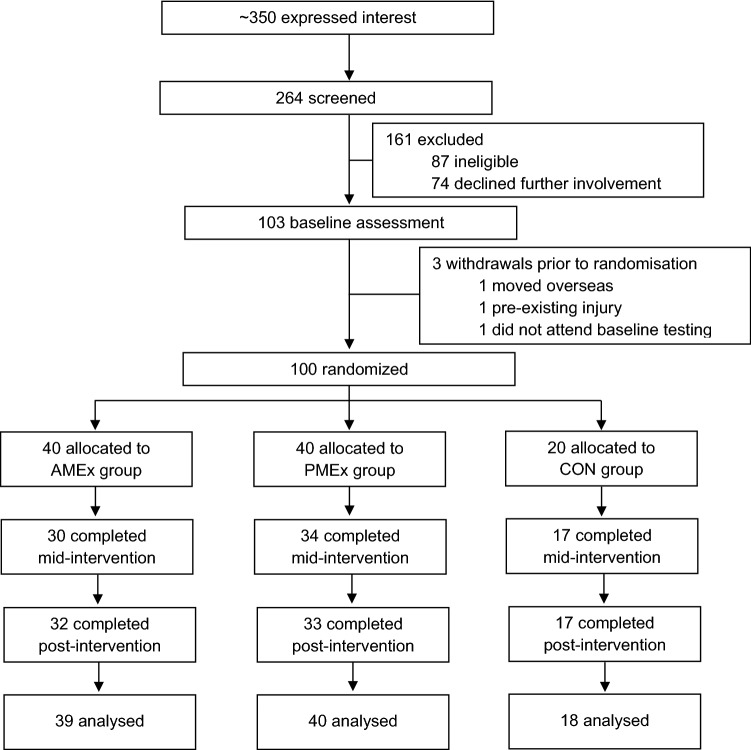
CONSORT flow diagram of participant progression through the study *Abbreviations:* AMEx, morning exercise; PMEx, evening exercise; CON, control

Ninety-seven participants had ≥ 2 time use profiles recorded at baseline and were included in the analysis (Fig. 1). In accordance with the CONSORT statement, significance testing of baseline differences was not performed (Boer et al., 2015; Schulz et al., 2010). Demographic characteristics appear to be similar between groups; mean age and body mass index were 41 ± 12, 38 ± 11, and 38 ± 10 years, and 31.1 ± 4.3, 32.0 ± 5.9, and 29.3 ± 3.6 kg·m^−2^, respectively. Seventy-six per cent of participants were female, most (77%) were in full-time employment, and university-educated (83%; Table 1).

**Table 1:** Baseline demographic characteristics

	AMEx	PMEx	CON	Whole sample
n	39	40	18	98
% Female	73	75	85	76
Age, years^a^	41 ± 12	38 ± 11	38 ± 10	39 ± 11
Weight, kg^a^	88.47 ± 11.6	90.86 ± 18.6	84.80 ± 12.6	88.69 ± 15.0
BMI, kg·m^−2a^	31.06 ± 4.3	31.99 ± 5.9	29.29 ± 3.6	31.08 ± 4.9
Education level				
Did not complete school	1	1	1	3
High school	1	3	0	4
Vocational qualification	5	4	1	10
University degree	33	32	18	83
Employment status				
Full-time	31	32	14	77
Part-time	6	2	5	13
Casual	1	5	0	6
Retired/unemployed	2	1	1	4
Marital status				
Married/de facto	30	26	13	69
Single/widowed	10	14	7	31
Dependents				
Yes	18	14	9	41
No	22	26	11	59

Data are reported as the number of participants

^a^Data are presented as Mean ± Standard Deviation

AMEx, morning exercise; PMEx, evening exercise; CON, control; BMI, body mass index

The average time (minutes/day) spent in each time use Superdomain across all timepoints for all groups is shown in Table 2. To be considered eligible to participate in this study, participants who enrolled were not meeting the physical activity guidelines of 150–300 min·wk^−1^ of moderate intensity activity / 75–150 min·wk^−1^ of vigorous activity, or an equivalent combination of both (Australian Government Department of Health & Ageing, 2014). According to baseline estimates, on average, participants were spending less than 70 min·wk^−1^ being physically active (Table 2). Time spent engaging in Physical Activity was significantly increased from baseline at both mid- (14–22 min·day^−1^) and post-intervention (12–19 min·day^−1^), for AMEx and PMEx. By mid-intervention, individuals in both exercise groups were meeting physical activity guidelines (AMEx, 154 min·wk^−1^; PMEx, 196 min·wk^−1^) and activity levels remained higher than baseline values at post-intervention (AMEx, 189 min·wk^−1^; PMEx, 126 min·wk^−1^). Although there were no significant differences in time spent engaging in PA between AMEx and PMEx during the intervention, there were some subtle differences which are worth acknowledging. Participants in AMEx continued to increase, albeit non-significantly, the time they spent in PA between mid- and post-intervention (21.9 versus 27.3 min·day^−1^, respectively; *p* = 0.330). In contrast, there was a decline in PA between mid- and post-intervention in the PMEx group (28.1 versus 17.7 min·day^−1^, respectively; *p* = 0.049). Relative to baseline values, time spent in Active Transport was also significantly increased at mid-intervention (16–19 min·day^−1^) for intervention groups, but these changes were no longer significant from baseline by the end of the intervention (Table 2).

**Table 2:** Use-of-time (min·day^−1^) during the intervention, measured by MARCA: within-group changes

Outcome	Group	Baseline (0 week)		Mid (6 week)			Post (12 week)			
		Mean	95% CI	Mean	95% CI	*p* (0 v. 6)	Mean	95% CI	*p* (0 v. 12)	*p* (6 v. 12)
PA	AMEx	8.2	2 to 15	21.9	14 to 30	**0.008**	27.3	19 to 36	** < 0.001**	0.330
	PMEx	6.0	− 1 to 13	28.1	30 to 36	** < 0.001**	17.7	10 to 26	**0.026**	**0.049**
	CON	9.6	0 to 19	8.4	− 2 to 19	0.859	5.5	− 6 to 17	0.575	0.689
Computer	AMEx	229.5	191 to 268	234.9	189 to 281	0.822	222.7	177 to 269	0.787	0.647
	PMEx	254.6	216 to 293	221.8	179 to 265	0.149	231.9	187 to 276	0.346	0.684
	CON	236.2	183 to 290	232.0	172 to 292	0.895	206.2	142 to 270	0.385	0.459
Active Transport	AMEx	34.4	26 to 43	50.0	40 to 40	**0.006**	43.8	33 to 54	0.143	0.313
	PMEx	35.0	27 to 43	53.1	44 to 63	**0.001**	44.7	35 to 55	0.120	0.141
	CON	30.4	19 to 42	30.5	17 to 44	0.981	33.1	19 to 48	0.755	0.749
Passive Transport	AMEx	80.3	67 to 94	83.9	68 to 99	0.633	83.0	67 to 99	0.749	0.920
	PMEx	86.7	73 to 100	85.6	71 to 100	0.878	91.8	76 to 107	0.548	0.430
	CON	65.9	47 to 85	56.2	36 to 77	0.333	74.5	52 to 97	0.476	0.100
Quiet Time	AMEx	72.9	54 to 92	61.3	39 to 83	0.333	58.9	37 to 81	0.226	0.849
	PMEx	61.5	43 to 80	62.9	42 to 84	0.900	65.5	44 to 87	0.722	0.833
	CON	60.4	35 to 86	64.0	35 to 93	0.821	61.7	31 to 92	0.940	0.892
Self-care	AMEx	103.2	96 to 111	113.8	105 to 122	**0.022**	109.1	101 to 117	0.149	0.349
	PMEx	108.1	101 to 115	113.9	106 to 122	0.191	106.9	99 to 115	0.755	0.140
	CON	100.5	91 to 110	106.0	95 to 117	0.361	96.2	85 to 108	0.448	0.139
Socio-cultural	AMEx	106.6	85 to 128	93.0	67 to 119	0.336	104.8	79 to 131	0.896	0.446
	PMEx	108.2	87 to 130	100.6	76 to 125	0.567	99.5	75 to 124	0.512	0.938
	CON	88.1	58 to 118	86.1	52 to 120	0.915	98.5	63 to 134	0.583	0.543
Work and study	AMEx	56.0	33 to 79	46.7	19 to 74	0.559	41.1	13 to 69	0.400	0.750
	PMEx	59.5	37 to 82	76.1	51 to 102	0.269	72.1	45 to 99	0.459	0.809
	CON	40.8	10 to 72	53.7	18 to 89	0.540	44.7	6 to 83	0.873	0.699
Chores	AMEx	163.2	135 to 192	185.3	153 to 218	0.147	158.3	125 to 192	0.772	0.110
	PMEx	136.3	108 to 165	123.6	93 to 155	0.379	128.3	96 to 161	0.624	0.768
	CON	153.3	114 to 193	184.5	141 to 228	0.120	160.1	114 to 206	0.770	0.272
Sleep	AMEx	468.0	447 to 489	474.3	450 to 499	0.627	502.8	478 to 527	**0.010**	**0.048**
	PMEx	469.6	449 to 490	489.0	466 to 512	0.116	495.7	472 to 520	**0.043**	0.612
	CON	497.9	469 to 527	495.1	463 to 527	0.869	512.5	479 to 547	0.423	0.356
TV/Videogames	AMEx	117.8	93 to 142	80.8	51 to 110	**0.029**	89.6	61 to 118	**0.032**	0.626
	PMEx	113.8	89 to 138	83.0	55 to 111	0.054	84.5	57 to 112	**0.022**	0.933
	CON	156.9	123 to 191	119.3	81 to 158	0.089	140.4	101 to 179	0.361	0.373

Significant changes are shown in bold. Data presented are estimated marginal means

MARCA, multimedia activity recall for children and adults; AMEx, morning exercise; PMEx, evening exercise; CON, control; Mid, mid-intervention, Post, post-intervention; CI, confidence interval; PA, physical activity; TV, television

For AMEx, time spent in Self Care significantly increased by 11 min·day^−1^ at mid-intervention, but this was no longer significantly different from baseline at post-intervention. At the end of the intervention, participants reported spending more time engaging in Physical Activity and Sleep (12–19 min·day^−1^, and 20–36 min·day^−1^, respectively), relative to baseline. To accommodate this time, a significant decrease was seen in the Television/Videogames domain (25–32 min·day^−1^). There were no significant changes in the time participants spent: using the Computer, in Passive Transport, in Quiet Time, performing Chores, in Work and Study, or in Socio-Cultural activites in either AMEx or PMEx.

**Fig. 2:**
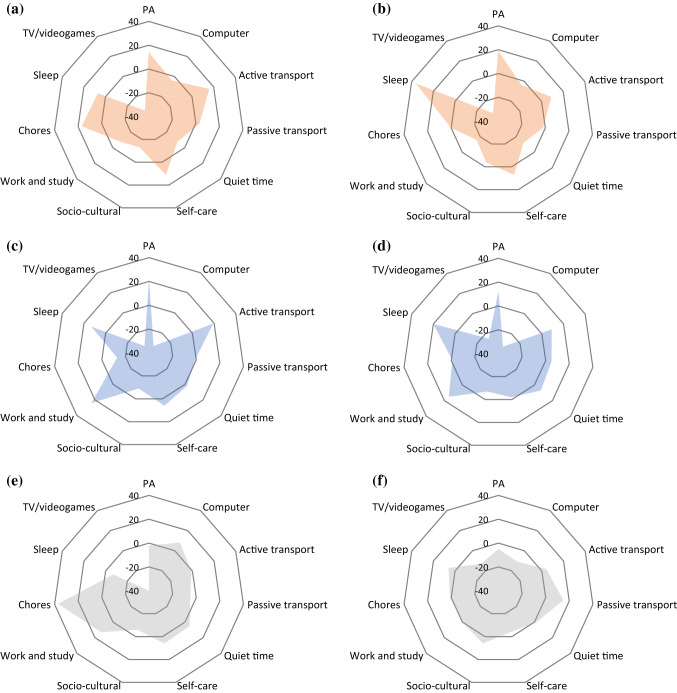
Change in time (min·day^−1^) spent in the 11 Superdomains, relative to baseline, measured by MARCA. (A) AMEx, mid-intervention; (B) AMEx, post-intervention; (C) PMEx, mid-intervention; (D) PMEx, post-intervention; (E) CON, mid-intervention; (F) CON, post-intervention. *Note* The radar chart use a radial display of the 11 Superdomains on different quantitative axes. Each axis represents a quantitiat for each superdomain. Data plotted on the zero axis represents the time spent (min·day^−1^) in that superdomain is equal to the baseline value. Positive values (i.e., > 0) indicate more time was spent in that superdomain compared with baseline, and negative values (i.e., < 0) indicate less time was spent in that superdomain compared with baseline.* Abbreviations* MARCA, multimedia activity recall for children and adults; AMEx, morning exercise; PMEx, evening exercise; CON, control; PA, physical activity; TV, television; min: minutes

There were no significant changes observed in any of the 11 time use Superdomains during the intervention for CON at either mid- or post-intervention assessment. Figure 2 illustrates the shifts in time use among Superdomains in all groups, relative to baseline values.

In response to the intervention, the patterns of change in AMEx and PMEx were similar, and both AMEx and PMEx were statistically different from CON for time spent in Physical Activity, Active Trasnport, Passive Transport and TV/Videogames (Table 3).

**Table 3:** Use-of-time (min·day^−1^) during the intervention, measured by MARCA: between-group differences

Outcome	Time	AMEx v. CON			PMEx v. CON			AMEx v. PMEx		
		Mean	95% CI	p	Mean	95% CI	p	Mean	95% CI	p
PA	Baseline	− 1.4	− 13 to 10	0.812	− 3.6	− 15 to 8	0.536	2.2	− 7 to 12	0.646
	Mid	13.4	0 to 27	0.051	19.7	7 to 33	**0.003**	− 6.3	− 18 to 5	0.275
	Post	21.9	8 to 36	**0.003**	12.2	− 2 to 26	0.086	9.6	− 2 to 21	0.100
Computer	Baseline	− 6.7	− 73 to 60	0.842	18.5	− 48 to 85	0.581	− 25.2	− 80 to 30	0.365
	Mid	2.8	− 73 to 78	0.941	− 10.2	− 84 to 64	0.787	13.0	− 50 to 76	0.684
	Post	16.6	− 62 to 95	0.679	25.7	− 52 to 104	0.516	− 9.1	− 73 to 55	0.780
Active transport	Baseline	4.0	− 11 to 19	0.589	4.6	− 10 to 19	0.535	− 0.6	− 13 to 11	0.924
	Mid	19.4	3 to 36	**0.023**	22.5	6 to 39	**0.007**	− 3.1	− 17 to 11	0.659
	Post	10.6	− 7 to 28	0.240	11.5	− 6 to 29	0.197	− 0.9	− 15 to 14	0.903
Passive transport	Baseline	14.4	− 9 to 37	0.221	20.8	− 2 to 44	0.075	− 6.5	− 25 to 13	0.504
	Mid	27.7	2 to 54	**0.036**	29.5	4 to 55	**0.023**	− 1.7	− 23 to 20	0.874
	Post	8.5	− 19 to 36	0.538	17.3	− 10 to 44	0.208	− 8.8	− 31 to 13	0.438
Quiet time	Baseline	12.5	− 19 to 44	0.436	1.0	− 30 to 33	0.949	11.5	− 15 to 38	0.386
	Mid	− 2.7	− 39 to 34	0.885	− 1.1	− 37 to 34	0.950	− 1.5	− 32 to 29	0.920
	Post	− 2.8	− 40 to 35	0.883	3.8	− 33 to 41	0.840	− 6.6	− 37 to 24	0.670
Self-care	Baseline	2.7	− 9 to 15	0.654	7.7	− 4 to 20	0.205	− 5.0	− 15 to 5	0.322
	Mid	7.8	− 6 to 22	0.264	7.8	− 6 to 22	0.253	0.0	− 12 to 12	0.999
	Post	13.0	− 1 to 27	0.069	10.7	− 3 to 25	0.127	2.3	− 9 to 14	0.700
Socio-cultural	Baseline	18.5	− 19 to 57	0.326	20.1	− 17 to 57	0.284	− 1.6	− 32 to 29	0.917
	Mid	6.9	− 36 to 50	0.750	14.5	− 27 to 43	0.494	− 7.6	− 43 to 28	0.675
	Post	6.3	− 38 to 50	0.779	1.0	− 43 to 45	0.965	5.3	− 31 to 41	0.770
Work and study	Baseline	15.2	− 24 to 54	0.440	18.6	− 20 to 57	0.830	− 3.5	− 35 to 28	0.830
	Mid	− 7.0	− 52 to 38	0.761	22.4	− 22 to 66	0.315	− 29.4	− 67 to 8	0.124
	Post	− 3.6	− 51 to 44	0.882	27.4	− 19 to 74	0.250	− 31.0	− 70 to 8	0.114
Chores	Baseline	9.9	− 39 to 59	0.690	− 17.0	− 66 to 32	0.491	26.9	− 13 to 67	0.190
	Mid	0.8	− 53 to 55	0.977	− 60.9	− 114 to − 8	**0.025**	61.7	17 to 107	**0.008**
	Post	− 1.9	− 59 to 55	0.949	− 31.9	− 88 to 24	0.265	30.0	− 16 to 76	0.949
Sleep	Baseline	− 30.0	− 65 to 5	0.096	− 28.3	− 64 to 7	0.114	− 1.7	− 31 to 28	0.911
	Mid	− 20.9	− 61 to 20	0.310	− 6.1	− 46 to 33	0.760	− 14.7	− 48 to 19	0.389
	Post	− 9.8	− 52 to 32	0.647	− 16.8	− 58 to 25	0.425	7.0	− 27 to 41	0.684
TV/Videogames	Baseline	− 39.1	− 81 to 3	0.067	− 43.2	− 85 to − 2	**0.043**	4.1	− 31 to 39	0.816
	Mid	− 38.5	− 87 to 10	0.120	− 36.3	− 84 to 11	0.134	− 2.2	− 43 to 38	0.915
	Post	− 50.9	− 99 to − 3	**0.038**	− 56.0	− 104 to − 8	**0.021**	5.1	− 34 to 44	0.797

Significant differences are shown in bold. Data presented are estimated marginal means

MARCA, multimedia activity recall for children and adults; AMEx, morning exercise; PMEx, evening exercise; CON, control; Mid, mid-intervention, Post, post-intervention; CI, confidence interval; PA, physical activity; TV, television

## Discussion

How individuals restructure the timing of their behaviors can influence the effectiveness of exercise, and have important health consequences, depending on what activities are displaced (Chastin et al., 2015; Gomersall et al., 2014; Mekary et al., 2009, 2013; Olds et al., 2012). The objective of this study was to investigate how previously inactive adults restructure their time when they undertake morning or evening exercise. Time spent engaging in physical activity was significantly increased from baseline at both mid- and post-intervention for AMEx and PMEx. There were some significant shifts in time use during the intervention period, and the patterns of change were similar between the intervention groups. Participants reported more time spent sleeping, and less time watching television/playing videogames in both AMEx and PMEx. The shifts in time use in this study likely have important health benefits.

According to baseline estimates, on average, participants spent approximately two hours per day watching television (range 114 to 157 min·day^−1^). In this study, the addition of exercise, prescribed at a volume of ≥ 250 min·wk^−1^ largely displaced time previously spent sedentary (i.e., watching television). In the intervention groups, Television viewing decreased by approximately 30 min·day^−1^. This would appear to be an important reduction; Stamatakis and colleagues estimate that watching two or more hours of television per day increases cardiovascular disease risk by 125% (Stamatakis et al., 2011).

Both intervention groups reported an increase in Active Transport at mid-intervention and higher levels of both Active Transport compared with participants in the control group. However, these changes and differences were no longer significant post-intervention. By design, the number of supervised exercise sessions reduced from mid- (4 sessions·wk^−1^) to post-intervention (2 sessions·wk^−1^), which may explain this finding (i.e., individuals did not have to travel to the exercise venue as frequently). These findings suggest that active transport may be an added benefit to supervised sessions beyond the prescribed exercise alone. Higher levels of active transport have also been associated with cardiometabolic and nutritional benefits including lower BMI, lower waist circumference, lower cholesterol and higher vitamin D (Passi-Solar et al., 2020). Without examining intervention effects using a time use approach, the nuanced ripple effects of changes in time would not be identified and the additional understanding which can be used to adapt future research would be unrealised.

Regular exercise is advocated to improve sleep quality, either by accelerating sleep onset or increasing the depth of sleep, and is routinely included in sleep hygiene recommendations (Buman & King, 2010; Chennaoui et al., 2015). However, it has been suggested that exercising close to retiring to bed may disrupt sleep, and thus, previous sleep hygiene recommendations have been to exercise 5–6-h before bedtime, and avoid activity for 3-h before bed (Morin et al., 1134; Schutte-Rodin et al., 2008). This is unsubstantiated, and even contradicted in experimental trials (Benloucif et al., 2004; Buman & King, 2010; Buman et al., 2014; Larsen et al., 2019; Yoshida et al., 1998). In support of the more recent evidence, with the addition of exercise, participants in AMEx and PMEx reported more time spent sleeping (AMEx, + 36 min·day^−1^; PMEx, + 20 min·day^−1^). Short sleep duration has been associated with obesity risk; a meta-analysis of short sleep duration, including 604,509 adults, reported that, for a one hour reduction in sleep, there was an increase in BMI of 0.35 kg·m^−2^ (Cappuccio et al., 2008). According to baseline estimates, participants in our study had an average sleep duration of more than seven hours (range 7.8 to 8.3 h·day^−1^). Therefore, the increase in sleep duration reported in our study during the intervention is unlikely to be of clinically significance.

This is the first study to examine changes in use-of-time in response to an exercise intervention prescribed at specific times of the day. In response to a 12-week exercise program performed in the morning (0600–0900) and the evening (1600–1900), we found no difference in how previously insufficiently active individuals spend, or reorganise their time to accommodate the new activity. Gomersall and colleagues investigated the changes in use-of-time across a 6-week physical activity intervention prescribed either 150 min·wk^−1^ or 300 min·wk^−1^ (Gomersall et al., 2014). The researchers found that time to accommodate increased physical activity was largely drawn from time spent watching television. However, there were no differences in patterns of change in time use between the intervention groups. Taken together, these findings suggest that, regardless of the dose or timing of exercise prescribed, the activities that people shift (or swap) to accommodate a short-term change in exercise participation are similar.

Traditionally, time use surveys have been used to capture data such as, how respondents spend their ‘free time’; residual time that remains after accounting for time spent in ‘paid labour’, ‘unpaid household labour’ and ‘personal care’. As in the case for the Time Use Survey conducted by the Australian Bureau of Statistics, respondents are asked to keep a diary of their daily activities for two consecutive days (Goodin et al., 2002). However, these surveys have been used to capture a population-level insight into how individuals use their time, rather than tracking change in time use in response to an intervention or stimulus, and require additional coding by the analysts. Therefore, a key strength of this study is the use of the MARCA, a validated, reliable, high-resolution 24-h recall tool, which enabled a comprensive examination of participants use of time (Gomersall et al., 2010). However, due to the self-reported nature of the tool, we cannot discount social desirability and recall bias. There are also some limits to the generalisability of our findings. The sample included in this study was one of convenience, predominantly recruited from students and staff at a metropolitan university, and with a bias toward females. This study was a pre-planned secondary analysis conducted within a larger randomized controlled trial aimed at investigating the influence of time-of-day of exercise on cardiometabolic health (Brooker et al., 2019). Thus, the study was not powered to detect statistical differences between groups for secondary outcomes, or an interaction over time. Therefore, null outcomes should be treated with caution and require replication. This study is also limited by its short, 12-week intervention duration. While the use of the MARCA minimized bias by requiring individuals to recount their whole of day (24-h) activity, we cannot rule out social desirability and recall bias (Althubaiti, 2016). Finally, no follow-up data on participants’ use of time after the cessation of the intervention were collected so it is unknown whether participants maintained their exercise, and if they continued to train at the prescribed times of day.

Patterns of time use present a novel way of examining the ripple effects of changes in daily activity patterns to accommodate for the time cost of exercise. This study used a randomized controlled trial to investigate how previously inactive adults restructure their time when they undertake morning or evening exercise. The time for exercise was larglely drawn from a discretionary time (watching TV), and the patterns of change in time use was similar when exercise was performed in the morning compared with the evening.

## Supplementary Information

Below is the link to the electronic supplementary material.

Supplementary file1 (DOCX 53 kb)

## Data Availability

The datasets generated during and/or analysed during the current study are available from the corresponding author on reasonable request.

## Abbreviations

AMEx: Morning exercise
CON: Control
PMEx: Evening exercise
