# Supplementary material for: How do previously inactive individuals restructure their time to ‘fit in’ morning or evening exercise: a randomized controlled trial
Source: J Behav Med. 2022 Nov 3;46(3):429–39. doi: 10.1007/s10865-022-00370-x (PMC10261239; doi:10.1007/s10865-022-00370-x)
Supplement: Supplementary file 1 — Supplementary file1 (DOCX 53 kb) [file 10865_2022_370_MOESM1_ESM.docx]

Supplementary Table A1. Tools used to promote behavior change

| Theoretical component | Description |
| --- | --- |
| Self-efficacy | **Self-monitoring strategies**  Participants recorded their behavior using exercise diaries. The act of keeping daily records requires conscious thought about activity levels and serves as a reminder to exercise [44].  Additionally, participants weighed themselves weekly to promote weight loss and reduce the risk of weight regain [45, 46]. |
| Goal setting | Participants were taught goal-setting techniques and instructed to set at least one goal specific to exercise. Goals and progress were reviewed by the participant and principal investigator every three weeks. |
| Relapse prevention | Problem-solving and coping strategies were addressed by discussion [47, 48]. |
| Outcome expectancy | Participants were given realistic expectations of the potential benefits of exercise, such as improved fitness and weight loss, to reduce rates of attrition [49, 50]. |
| Education and skill development | Participants were given instructions, feedback and guidance about the use of exercise equipment, exercise technique, and recognising cues for injury avoidance.  Participants were also familiarised with basic concepts in nutrition such as the Australian Dietary Guidelines and portion size control [51] and encouraged to visit the UQ St Lucia Dietetics Practice. |
| Prompting | Participants were sent email/text message reminders to attend their supervised exercise sessions. After their session, participants were also prompted to incorporate some active transport or active leisure time each week to meet the required dose of unsupervised exercise. |
| Encouragement and support | Participants were asked about their progress and any potential barriers to exercise were identified and methods to help overcome them were discussed. |
| Environmental | During the supervised exercise sessions, music was played to improve mood and increase motivation and exercise performance [52]. |

*Abbreviation:* UQ, University of Queensland.

**Supplementary Table A2. The 11 time-use superdomains used in the study**

| Superdomain | Description | Examples |
| --- | --- | --- |
| Sleep | All sleep including naps |  |
| Quiet time | Time spent without interaction | Reading  Listening to music |
| Active transport | Walking or cycling | Climbing stairs  Walking |
| Passive transport | Riding or driving motorised transport | Driving a car  Riding in a bus |
| Physical activity | Exercise and sport | Lifting weights  Tennis |
| Work and study | Occupational or study-related activities | Clerical work  Homework |
| Computer | Using a computer except for videogames | Email  Internet |
| TV/Videogames | Watching TV and playing videogames | Watching TV  X-Box |
| Self-care | Eating and grooming | Showering  Having dinner |
| Chores | Indoor and outdoor domestic chores | Food preparation  Hanging out washing |
| Socio-cultural | Arts and crafts | Playing a musical instrument  Card games |

*Note:* Adapted from “In search of lost time: When people undertake a new exercise program, where does the time come from? A randomized controlled trial,” [Supplemental material] by S.R. Gomersall et al., 2014, *Journal of Science and Medicine in Sport, 18*, p. 1. [8].

*Abbreviation:* TV, television.
